# HEX expression and localization in normal mammary gland and breast carcinoma

**DOI:** 10.1186/1471-2407-6-192

**Published:** 2006-07-19

**Authors:** Cinzia Puppin, Fabio Puglisi, Lucia Pellizzari, Guidalberto Manfioletti, Marta Pestrin, Maura Pandolfi, Andrea Piga, Carla Di Loreto, Giuseppe Damante

**Affiliations:** 1 Dipartimento di Scienze e Tecnologie Biomediche, Università di Udine, Italy; 2 Dipartimento di Scienze Mediche e Morfologiche, Università di Udine, Italy; 3 Istituto di Genetica del Policlinico Universitario, Università di Udine, Italy; 4 Dipartimento di Biofisica, Biochimica e Chimica delle Macromolecole, Università di Trieste, Italy; 5 Associazione Ricerca Traslazionale In Senologia

## Abstract

**Background:**

The homeobox gene HEX is expressed in several cell types during different phases of animal development. It encodes for a protein localized in both the nucleus and the cytoplasm. During early mouse development, HEX is expressed in the primitive endoderm of blastocyst. Later, HEX is expressed in developing thyroid, liver, lung, as well as in haematopoietic progenitors and endothelial cells. Absence of nuclear expression has been observed during neoplastic transformation of the thyroid follicular cells. Aim of the present study was to evaluate the localization and the function of the protein HEX in normal and tumoral breast tissues and in breast cancer cell lines.

**Methods:**

HEX expression and nuclear localization were investigated by immunohistochemistry in normal and cancerous breast tissue, as well as in breast cancer cell lines. HEX mRNA levels were evaluated by real-time PCR. Effects of HEX expression on Sodium Iodide Symporter (NIS) gene promoter activity was investigated by HeLa cell transfection.

**Results:**

In normal breast HEX was detected both in the nucleus and in the cytoplasm. In both ductal and lobular breast carcinomas, a great reduction of nuclear HEX was observed. In several cells from normal breast tissue as well as in MCF-7 and T47D cell line, HEX was observed in the nucleolus. MCF-7 treatment with all-trans retinoic acid enhanced HEX expression and induced a diffuse nuclear localization. Enhanced HEX expression and diffuse nuclear localization were also obtained when MCF-7 cells were treated with inhibitors of histone deacetylases such as sodium butyrate and trichostatin A. With respect to normal non-lactating breast, the amount of nuclear HEX was greatly increased in lactating tissue. Transfection experiments demonstrated that HEX is able to up-regulate the activity of NIS promoter.

**Conclusion:**

Our data indicate that localization of HEX is regulated in epithelial breast cells. Since modification of localization occurs during lactation and tumorigenesis, we suggest that HEX may play a role in differentiation of the epithelial breast cell.

## Background

The homeobox gene HEX (known also as Prh) encodes for a tissue-specific transcription factor that plays a role during various phases of vertebrate development [1]. It binds DNA in a sequence-specific manner and is able either to activate or repress transcription of target genes [2]. During early mouse development, HEX is first expressed in the primitive endoderm of blastocyst and, after unilateral cell movements, it marks the anterior visceral endoderm [3]. Later, HEX is expressed in developing thyroid, liver, lung, as well as in haematopoietic progenitors and endothelial cells [3,4]. Disruption of HEX gene results in embryonic lethality due to block of early liver development [1,5]. Moreover, HEX-null mice shows defects in forebrain and thyroid as well as in differentiation of the monocyte lineage. Although HEX is primarily known as a transcriptional regulator [6], in several situations HEX is localized primarily in the cytoplasm. For example, HEX localization is nuclear in endodermal cells that give rise to the liver, while it is cytoplasmic in cells lateral to the liver-forming region [7]. Moreover, in malignant thyroid tumors, HEX expression is confined to the cytoplasm only [8]. Accordingly, it has been demonstrated that HEX is able to interact with factors that have cytoplasmic functions such as proteasome proteins [9] and eIF4E [10]. Misexpression of HEX gene may have a causal role in neoplastic cell proliferation. In fact, HEX overexpression in haemopoietic precursor cells promotes development of T-cell-derived lymphomas [11]. It has recently been shown that HEX protein is transiently expressed during development of skin and that its overexpression in dermal fibroblasts stimulates proliferation of epidermal cells [12]. Thus, most likely, HEX gene is expressed and plays a functional role in several additional cell types beyond those identified in early investigations.

Breast cancer is the leading cause of cancer death in women worldwide [13]. A better understanding of the molecular mechanisms involved in breast cancer formation and progression is therefore of crucial importance. To date, no studies have been carried out to evaluate the role of HEX gene in breast cells. In the present study, we have investigated the expression of HEX protein in normal and cancerous breast tissue as well in breast cancer cell lines.

## Methods

### Tissue samples and cell lines

The present study included: 9 normal, non-lactating breast tissues; 3 lactating breast tissues; 14 ductal breast carcinomas and 6 lobular breast carcinomas. Donor patients received no preoperativechemotherapy or hormonotherapy. MCF-7 and T47D cell lines were cultured in DMEM supplemented with 10% fetal bovine serum (Gibco). HBL 100 cell line was cultured in RPMI with 10% fetal bovine serum (Gibco). The study was conducted in accordance with the tenets of the Declaration of Helsinki. Following the indication of Italian DLgs no. 196/03 (Codex on Privacy) a written consent was obtained from all patients.

### Immunohistochemistry

Formalin-fixed, paraffin-embedded samples were evaluated for the expression of HEX protein using an immunoperoxidase technique. Sections of formalin-fixed, paraffin embedded representative blocks of breast cancer were cut onto silane-coated slides and dewaxed. After blocking of endogenous peroxidase, sections were incubated with rabbit antiserum to HEX diluted 1:250 in PBS overnight at 4°C. After washing, the peroxidase EnVision System (HPR rabbit/Mouse Envision System TM, DakoCytomation, Denmark) was applied for 30 min at room temperature. Peroxidase activity was detected with 3,3'-diaminobenzidine tetrachloride (Sigma) and haematoxylin counterstain. Negative controls were carried out by replacing the primary antiserum with pre-immune rabbit serum. The HeLa cell line, which does not expresses HEX [14], has been used as negative control. The specificity of antiserum to HEX was previously described [8]. A brown staining in the nucleus and/or in the cytoplasm indicated the presence of HEX protein. The HEX expression was evaluated calculating separately nuclear and cytoplasmic reactivity in 1000 cells, and was expressed as a percentage of positive cells. In breast cancer samples an immunohistochemical evaluation of oestrogen and progesterone receptors (1D5 and 636 clone, DakoCytomation, Denmark) and proliferation fraction with ki-67 (MIB-1 clone DakoCytomation, Denmark) was done and expressed as percentage of positive nuclei. In the case of cell cultures (see below), cells were immediately fixed with ethanol containing 5% glacial acetic acid for 10 min at 4°C, and rinsed with 0.1% saponin (Sigma Chemical Co., St Louis, MO, USA) in PBS. This PBS-saponin solution was also used for all subsequent washing steps. The cultures were incubated overnight at 4°C with rabbit antiserum to HEX diluted 1:100. After washing, the peroxidase EnVision System (HPR rabbit/Mouse Envision System TM, DakoCytomation, Denmark) was applied for 30 min at room temperature.

Phosphatase alkaline was developed with new fuchsin (Fuchsin + substrate - Chromogen, Dako, Denmark) containing levamisole. Control staining used pre-immune rabbit serum (Dako A/S, Denmark) instead of the primary antiserum.

### Real-time PCR

Quantitative PCR analysis of HEX mRNA expression was performed as previously described [15]. Briefly, total RNA was extracted with RNeasy protect mini kit (Qiagen). 1 μg of total RNA was reverse-transcribed to single strand cDNA using random exaprimers and 200 U of Superscript II reverse transcriptase (Invitrogen, Milan, Italy) in a final volume of 20 μl, at 42°C for 50 min followed by heating at 70°C for 15 min.

Real time PCR reactions were performed using the ABI Prism 7300 Sequence Detection System (Applied Biosystems, Foster City, CA, USA). Oligonucleotide primers and probes for HEX were purchased from Applied Biosystems as Assays-on-Demand Gene Expression Products. Oligonucleotide primers and probe for the endogenous controls Abelson (ABL), Beta-glucuronidase (GUS) and Beta-2-microglobulin (B2M) are described by Beillard et al. [16]. A 25 μl reaction mixture containing 5 μl of cDNA template, 12.5 μl TaqMan Universal PCR master mix (Applied Biosystems) and 1.25 μl primer probe mixture was amplified using the following thermal cycler parameters: incubation at 50°C for 2 min and denaturation at 95°C for 10 min, then 40 cycles of the amplification step (denaturation at 95°C for 15 sec and annealing/extension at 60°C for 1 min). The ΔCT method, by means of the SDS software (Applied Biosystem), was used to calculate the mRNA levels.

### Cell transfection

The Sodium Iodide Symporter (NIS) gene promoter activity was investigated by using a clone, kindly provided by U. Loos (University of Ulm, Germany), containing 2.2 Kb of 5' genomic sequence for the NIS promoter, containing the minimal promoter linked to the luciferase (LUC) gene as reporter [17,18]. The HEX and PAX8 expression vector have been previously described [14,19].

The transfection efficiency was normalized by cotransfecting the CMV-βGAL plasmid, which contains the Cytomegalovirus (CMV) promoter linked to the β-galactosidase (βGAL) gene.

The calcium phosphate co-precipitation method used for transfections was performed as previously described [20]. HeLa cells were plated at 6 × 10^5 ^cells/100-mm culture dish 20 hours prior to transfection. Plasmids were used in the following amount per dish: CMV-βGAL, 2 μg; pNIS-LUC, 7 μg; HEX expression vector 1 μg and 2 μg, PAX8 expression vector 1 μg and 2 μg. In both HEX and PAX8 expression vectors transcription is driven by the CMV promoter. An empty vector containing the CMV promoter has been cotransfected with the reduced amount of the HEX and PAX8 expression vectors to avoid squelching effects. Cells were harvested 44 hours after transfection and cell extracts were prepared by a standard freeze-and-thaw procedure. βGAL protein was measured by ELISA method (Amersham Pharmacia Biotech, Milan, Italy). LUC activity was measured by a standard chemiluminescence procedure [21].

### Statistical analysis

Spearman's Correlation Coefficient Rho was used as a non-parametric measure of correlation between two ordinal variables. Statistical significancy was expressed as p < 0.05. Mann-Whitney and Kruskal-Wallis were performed as nonparametric alternatives to the one-way analysis of variance. Both tests look for differences among the populations medians.

## Results

**Figure 1:**
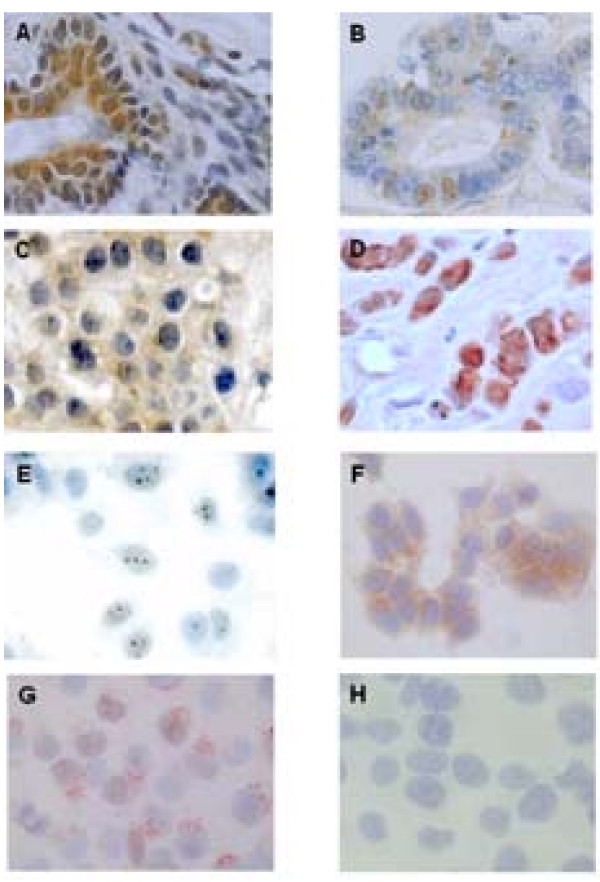
HEX expression and localization in normal tissues, breast carcinomas and cell lines. Immunohistochemical analysis was performed as described in the methods section. A and B: normal breast tissue. C: ductal carcinoma. D: lobular carcinoma. E: MCF-7 cells. F: T47D cells. G: HBL100 cells stained with p63 and CD10. H: HBL100 cells stained with HEX.

HEX expression was first evaluated by immunohistochemistry in 9 samples of normal breast tissue. As shown in panel A and B of Fig. 1, HEX was expressed in normal mammary gland. In particular, it was expressed in epithelial cells of the terminal duct-lobular unit. HEX was predominantly present in the cytoplasm with only a fraction of cells showing nuclear staining. In several epithelial cells a nucleolar reactivity was evident.

HEX expression in ductal and lobular breast carcinomas is shown in panels C and D of Fig. 1. The main characteristics of the analysed tumour samples are shown in Tab. 1 In breast carcinomas, HEX was mainly expressed in cytoplasm (median percentage of reactive cells: 70; 25^th^–75^th ^percentiles: 40–90) than in nucleus (median percentage of reactive cells: 0; 25^th^–75^th ^percentiles: 0–1.15). No significant differences were observed in terms of HEX nuclear or cytoplasmic staining intensity among the different areas of each tumour. Moreover, no staining intensity differences were observed among the different tumour samples. A significant difference between normal and cancer tissues, in terms of HEX nuclear reactivity, was present (Tab 2). According to the histotype, the nuclear HEX expression was higher in lobular (median 1.25%; 25^th^–75^th ^percentiles 0.6–2.1%) than in ductal (median 0%; 25^th^–75^th ^percentiles 0–0%) invasive carcinomas (p = 0.023). Higher expression of nuclear HEX was observed in grade 2 tumors than in the other grade categories (p = 0.05). This observation can be because all lobular carcinomas were grade 2. In fact, by excluding lobular invasive cancer from the analysis, no statistically significant association was found between grade and HEX nuclear staining (p = 0.7).

A correlation between HEX cytoplasmic expression and proliferative activity, evaluated by MIB-1 immunohistochemistry, just failed to reach significance (rho: 0.4, p = 0.07). A statistically significant direct correlation was found between estrogen receptor and HEX nuclear expression (rho = 0.6, p = 0.004). No statistically significant correlation was observed between HEX cytoplasmic staining and estrogen receptor expression (rho = 0.2, p = 0.25). In addition, there was a trend in favour of an inverse correlation between progesterone receptor expression and HEX cytoplasmic expression (rho: – 0.3, p = 0.09). No significant correlation was found between nuclear HEX staining and progesterone receptor expression (rho = -0.1, p = 0.9).

HEX expression was also investigated in mammary tumour cell lines. MCF-7 cells showed weak staining. In the nucleus, HEX was present predominantly at nucleolus level (Fig. 1, panel E). An evident nucleolar HEX localization was observed also in T47D cell line; in this cell line the cytoplasmic expression of HEX was more evident than that observed in MCF-7 cells (Fig. 1, panel F).

HBL100 are cells of myoepithelial origin [22]. In agreement to this notion, this line expressed well known markers of myoepithelial cells: p63 (in the nucleus) and CD10 (in the plasma membrane) (Fig. 1, panel G). HBL100 cells expressed HEX neither in the cytoplasm nor in the nucleus (Fig. 1, panel H). Altogether, these data would suggest that in mammary tissue HEX is expressed in epithelial and not in myoepithelial lineages.

**Figure 2:**
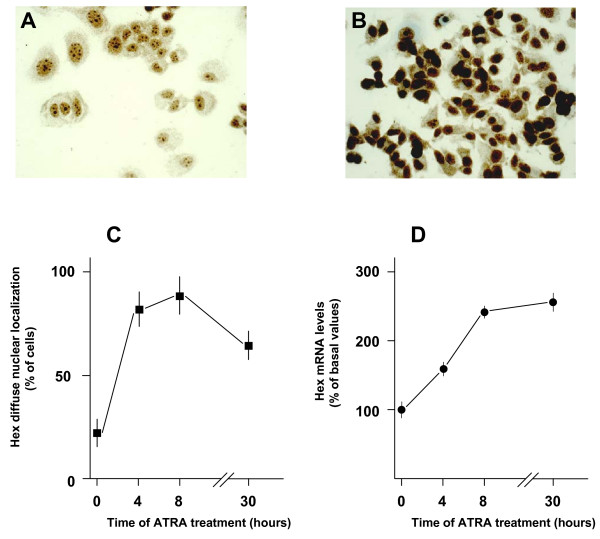
Effects of ATRA on HEX expression and localization in MCF-7 cells. HEX protein and HEX mRNA levels were evaluated by immunohistochemistry and real time PCR, respectively. A: untreated MCF-7 cells. B: MCF-7 cells treated for 15 hours with ATRA 1 μM. C: time-course of ATRA effect on HEX localization. Each square represents the mean value ± standard deviation of four 100 cells counts in different slides. D: time-course of ATRA effect on HEX mRNA levels. Each dot represents the mean value ± standard deviation of three independent determinations.

It has been recently demonstrated that in a vitamin-A deficient animal model, which lacks biologically active retinoic acid (RA), HEX expression is affected [23]. Thus, we tested whether RA is able to modify HEX expression/compartimentalization. MCF-7 cells were treated with all-*trans *RA (ATRA) for 15 hours at the concentration of 1 μM and HEX expression and localization was evaluated by immunohistochemistry. Fig. 2 (Panels A and B) shows that in the absence of ATRA a nucleolar positivity was preminent. The ATRA treatment induced an intense and diffuse nuclear positivity. Since differences between ATRA-treated and untreated cells were extremely evident, the time-course of ATRA action was assessed by measuring the percentage of cells with intense and diffuse nuclear localization with respect to those with only nucleolar positivity. As shown in panel C of Fig. 2, the effect of 1 μM ATRA was already evident after 4 hours treatment. In order to corroborate immunohistochemical data, HEX mRNA levels in MCF-7 cells were evaluated by real-time PCR before and after 1 μM ATRA treatment. As shown in panel D of Fig. 2, HEX mRNA levels increased upon ATRA treatment; as in the immunohistochemical assay, the ATRA effect was already evident after 4 hours treatment.

**Figure 3:**
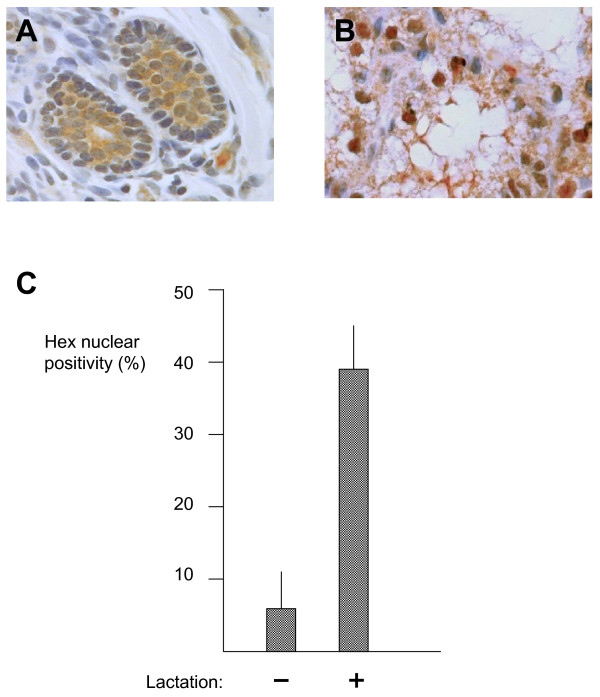
HEX nuclear localization in lactating breast. Panel A: non-lactating tissue. Panel B: lactating tissue. Panel C: quantification of the HEX nuclear positivity in non-lactating and lactating breast tissues. HEX nuclear positivity was evaluated by calculating separately nuclear and cytoplasmic reactivity in 1000 cells. Each bar represents the mean value ± standard deviation of nuclear HEX, expressed as percentage of stained cells in the nucleus.

Retinoids induce lumen morphogenesis in mammary epithelial cells [24]. Moreover, modification of HEX compartimentalization during differentiation has been previously observed [7]. Therefore, since the maximal differentiation state of the breast occurs during lactation, we tested the hypothesis that HEX nuclear localization could be modified in lactating mammary gland. HEX expression of five cases of non-lactating breast tissues and three cases of lactating breast tissues was assessed by immunohistochemistry in the same experiment. As shown in Fig. 3, the nuclear localization of HEX was greatly increased in nuclei of lactating breast cells. No significant differences were observed among the different cases of lactating breasts. These data suggest that HEX may play a role in controlling genes whose expression is modified during lactation.

**Figure 4:**
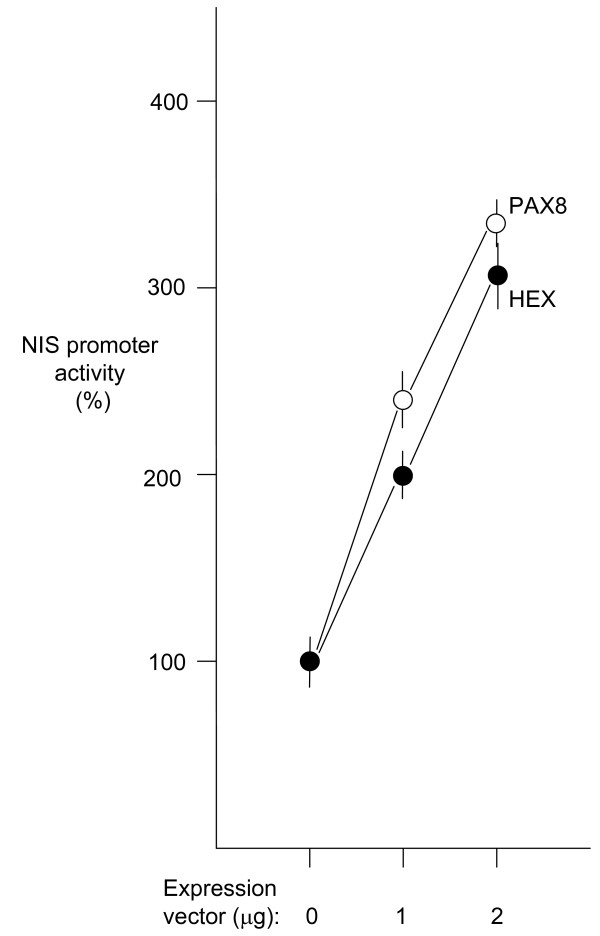
HEX effects on NIS promoter. HeLa cells were transfected with 1 and 2 μg of the HEX or PAX8 expression vector together with plasmids containing the NIS and the CMV promoters linked to the LUC and the βGAL reporter genes, respectively. The CMV-βGAL plasmid was used to normalize for efficiency of transfection. Each dot represents the mean value ± standard deviation of four independent transfections. Results are expressed as percentage of the values obtained in cells not transfected with HEX or PAX8 expression vectors.

The gene encoding NIS is expressed in mammary gland only during lactation [25]. Therefore, in order to assign a biological role to increased HEX nuclear localization during lactation, we tested whether HEX was able to activate NIS promoter. HeLa cells (which do not express the HEX gene), [14] were transfected with a construct containing the human NIS promoter linked to the LUC gene, either in presence or absence of expression vectors for HEX or PAX8. This latter is a known activator of NIS expression [19,26]. As shown in Fig. 4, the presence of HEX was associated to an increase of the NIS promoter activity, with a significant effect already at 1 μg of expression vector (p < 0.01, according to the Student's T test). The HEX effect was similar to that induced by PAX8 both at 1 and 2 μg. Thus, the overexpression of NIS during lactation could be, at least in part, due to the increase and relocalization of HEX.

**Figure 5:**
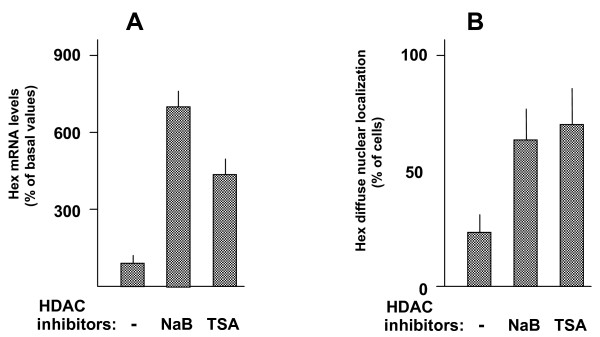
Effects of HDAC inhibitors on HEX expression and nuclear localization in MCF-7 cells. Cells were treated for 30 hours with NaB (3 mM) and TSA (300 nM). Then, HEX mRNA levels were evaluated by real-time PCR and HEX localization by immunohistochemistry. Panel A: effect of NaB and TSA on HEX mRNA levels. Panle B: effect of NaB and TSA on the HEX diffuse nuclear localization. In both panels each bar represents the mean value ± standard deviation of three independent measures. In both panels, according the paired Student's T test, the differences between treated and untreated cells were highly significant (at least: p < 0.01).

We recently reported that histone deacetylase (HDAC) inhibitors such as sodium butyrate (NaB) and trichostatin A (TSA) are able to increase NIS expression in MCF-7 cells [15]. Thus, in order to support the role of HEX in the control of NIS expression, MCF-7 cells were treated with NaB (3 mM) and TSA (300 nM) for 30 hours and subjected to real-time PCR and immunohistochemistry. Both compounds induced a diffuse nuclear localization, which was superimposable to that observed upon ATRA treatment (data not shown). As shown in Fig. 5, either compound increases HEX mRNA levels as well as the HEX diffuse nuclear localization. Therefore, the increase of NIS expression when MCF-7 cells are treated with NaB and TSA [15], is associated to the increase of HEX expression and of its diffuse nuclear localization, supporting the existence of a functional link between HEX and NIS in breast cells.

## Discussion

In this study we investigated the expression of HEX in normal and tumoral breast tissues as well as in breast cancer cell lines. As in other tissues, HEX is present both in nucleus and cytoplasm. In breast cells, however, it is extremely clear that, at the nuclear level, HEX is particularly evident in the nucleolus. Nucleoli are organelles within the nucleus that are assembled around clusters of tandemly repeated ribosomal genes (rDNA) [27]. They seem to be predominantly involved in ribosome biogenesis. There is, however, increasing evidence that nucleoli also have non-ribosomal functions, such as being the site for protein synthesis [28] and being involved in the regulation of proteins such as the tumor suppressor gene p53 [29], the proto-oncogene c-myc [30] and HIV-regulatory proteins [31,32]. It has been recently demonstrated that activation of HIF occurs by pH-dependent nucleolar sequestration of VHL [33]. Thus, the nucleolar localization of HEX, together with the cytoplasmic localization, could be part of a control mechanism to sequestrate HEX protein from its target genes.

Another interesting observation is that HEX nuclear localization is reduced in cancer cells. A similar phenomenon (decrease of HEX nuclear expression in cancer compared to normal tissue) has been previously observed in thyroid tumours [8]. These data suggest that the decrease of nuclear HEX in cell transformation could be a common phenomenon for tumours originating from HEX-expressing tissues. The decrease of HEX nuclear localization in tumours suggests that HEX compartimentalization could be related to the differentiation state of the cell. Accordingly, HEX nuclear localization is greatly increased during lactation.

HEX nuclear localization is greatly increased in lactating breast tissues and in MCF-7 cells treated with ATRA. Effects of vitamin-A deficiency on breast function and development are well documented. In rats, low vitamin-A intake affects expression of iron transporters and milk iron levels [34]. Vitamin-A deficient female mice show reduced ductal and lobular alveolar development and decrease of secretory activity [35]. Moreover, by using "in vitro" systems, it has been shown that RA promotes lumen formation by mammary epithelial cells [24]. It could be hypothesized, therefore, that nuclear HEX may play a role in breast cell differentiation and that this transcription factor is part of the molecular pathway by which RA controls development and function of the mammary gland.

We report several evidences indicating that the nuclear HEX levels may control NIS expression. First, HEX is able to activate the human NIS promoter at a similar extend of PAX8, which is a known activator of NIS expression [19,26]. Second, treatment of MCF-7 cells with 1 μM ATRA for 4 and 8 hours increases HEX expression and induces a diffuse nuclear localization. This effect fits perfectly well to the data reported by Kogai et al [36], which show that 1 μM ATRA treatment of MCF-7 cells induces increase of NIS expression, with HEX mRNA peaking after 12 hours. Third, treatment of MCF-7 cells with HDAC inhibitors induces HEX expression and its diffuse nuclear localization (see Fig. 5) as well as NIS expression [15]. It should pointed out, however, that the nuclear HEX is decreased in breast tumours, even though NIS is expressed [37]. It is possible that transcription factors different than HEX may contribute to NIS expression in breast tumours. In fact, it has been recently demonstrated that the transcription factor Nkx-2.5 is expressed in human breast cancer cells and is able to induce NIS expression [38]. HEX is expressed in thyroid follicular cells [14], therefore it may play a role in the control of NIS expression also in that cell type. The decrease of HEX nuclear localization that occurs in thyroid cancer cells [8], may contribute to the decrease of NIS expression that occurs in these neoplasms. Expression of the endogenous NIS gene in breast cancer may have a therapeutical relevance. In fact, NIS is responsible for uptake of iodide into cells. The presence of NIS in the basolateral membrane of thyroid follicular cells has been exploited for many years with the use of radioiodine to treat thyroid disease. The use of radiodine has proven to be a safe and effective way for imaging and treatment of thyroid cancer [39]. The finding that NIS gene is expressed in tissues other than the thyroid, has raised the possibility of using radioiodide for imaging and treatment of tumors of nonthyroid origin [40]. Our results may contribute to understand how to increase NIS expression in breast cancer.

## Conclusion

We have investigated the expression of HEX gene in human breast: as in other tissues, HEX is localized both in the nucleus and in the cytoplasm. HEX nuclear expression is down-regulated in breast cancer whilst it is up-regulated during lactation. In several cells from normal breast tissue as well as in MCF-7 and T47D cell line, HEX was observed in the nucleolus. Treatment of MCF-7 cells with ATRA increases both HEX expression and nuclear localization of the protein. These data indicate that localization of HEX is regulated in epithelial breast cells. Since modification of localization occurs during lactation and tumorigenesis, and is elicited by differentiation inducers, we suggest that HEX may play a role in differentiation of the epithelial breast cell. Data suggesting that HEX is able to increase the NIS promoter activity may indicate a molecular mechanism that could be potentially utilized to increase the expression of the endogenous NIS gene in breast cancer cells.

## Abbreviations

PCR: Polymerase Chain Reaction

NIS: Sodium Iodide Symporter

CMV: Cytomegalovirus

RSV: Rous Sarcoma Virus

LUC: Luciferase

CAT: Chloramphenicol acetyl transferase

RA: Retinoic Acid

ATRA: All Trans Retinoic Acid

## Competing interests

The author(s) declare that they have no competing interests.

## Authors' contributions

CP, cell biology experiments; FP, design of the study and statistical analysis; LP, real time PCR experiments; GM, antibody testing; MP, MP and CDL, selection of tissues, immunohistochemistry, evaluation of immunohistochemical results; AP, design of the study; GD, design and coordination of the study and writing the manuscript.

## Pre-publication history

The pre-publication history for this paper can be accessed here:



## Figures and Tables

**Table 1:** Characteristics of tumour samples

**Characteristic**	**N°**	**%**
Histotype:		
Ductal	14	70
Lobular	6	30
Grade:		
1	5	25
2	10	50
3	5	25
Hormonal receptor status:		
ER*positive	16	80
ER negative	4	20
PGR positive	11	55
PGR negative	9	45
HEX expression:		
Nuclear	7	35
Cytoplasmic	20	100

*: ER, Estrogen receptor; PGR, Progesterone receptor.

Positive samples were considered when specific staining was present in ≥ 10% of cells.

**Table 2:** HEX expression and localization in normal and breast carcinoma

**Tissues**	**Normal Median (25**^th^**–75**^th^**)**	**Cancer Median (25**^th^**–75**^th^**)**	**p value**
Nuclear HEX	2.5 (0.5–11.9)	0 (0–1.15)	0.008
Cytoplasmic HEX	70 (40–90)	78 (50–95)	NS

NS: not significant
